# Rank of the Medical Corps of the U. S. Army

**Published:** 1873-12

**Authors:** 


### Editorial.

### The Rank of the Medical Corps of the U. S. Army.

At the last meeting of the American Medical Association, a letter was received from the medical officers of the army regarding the rank of the medical corps in the army A committee was at once appointed, consisting of Drs. J. M. Keller, of Louisville, J. A. Murphy of Cincinnati, N. S. Davis, of Chicago, H. F. Askew, of Wilmington, Del.. and J. M. Toner, of Washington, to memorialize Congress, upon the subject. That the medical corps of the army has been treated with great injustice in the distribution of honors will be seen from the following brief abstract of the letters presented to the Association:

*To the President of the American Medical Association:*

SIR:—It is proposed to present herewith a brief statement, showing that the Medical Staff of the United States Army has not been placed on an equal footing with the other staff corps of the army as regards rank, that they have not had the same consideration shown them in this respect as has been accorded to the navy, and that the record of services of this meritorious body of officers entitles them to the same advantages that have been granted to others.

When the rebellion broke out, the Medical Staff of the army with a total of 115 officers, had but one of higher rank than that of Major, while in the

Quartermaster's Department, with 37 officers, 5 were above that rank; in the Commissary Department, with 12 officers. 2 were above that rank; in the Adjutant-General's Department, with 14 officers, 2 were above that rank, in the Engineer's, with 12 officers, there were 7; in the Pay Department, out of a total of 28, there were 3; and in the Ordnance, 2 out of 36.

The act of Congress of July 28, 1866, defined the "Peace establishment of the United States."

By its provisions the total number of medical officers were fixed at 217,7 of whom were above the rank of Major, or 3.22 per cent. The Quartermaster's Department was to consist of 76 officers, 17 of whom were above that rank, or over 22 per cent.; the Subsistence Department of 29 officers, 5 being above that rank, or 17 per cent; the Adjutant-General's Department, of 20 officers, 7 being above that rank, or 35 per cent.; the Engineers' of 109 officers, of whom 19 were above that rank, or over 17 per cent.; the Ordnance of 64 officers, with 8 above that rank, or 12.50 per cent.; and the Pay Department of 65 officers, 5 being above that rank, or 7.50 per cent. It is thus seen that the Medical Department was given a smaller proportion of officers of rank than any other staff department, being less than one-half of that granted to Paymasters, one quarter of that in the Ordnance, nearly one-sixth of that in the Engineers and Subsistence Departments, less than one-seventh of that in the Quartermasters, and about one-twelfth of that given to Adjutants General.

The medical officer under the present law is accorded the rank of Captain after three years' service; he is promoted to Major by seniority, such promotion not ordinarily occurring in less than fifteen years' service, and that is the end of his prospects of advancement, unless he may be so fortunate as to secure a Medical Purveyorship, which can necessarily be within the reach of but very few, those positions being but five in number, and vacancies in consequence occurring but very seldom. Not only is this a great injustice to those surgeons on the active list, but it operates still more injuriously to those old and meritorious officers, who, having given their best years to the country, become disabled by age or infirmity and are desirous of availing themselves o the privileges of the retired list. The Adjutant-General, Quartermaster, or Engineer of thirty years' service can almost certainly retire with the rank of Colonel, certainly with that of Lieutenant-Colonel, while the veteran Surgeon is laid aside with the rank of Major, and an income hardly sufficient to support his station as a gentleman, or to sustain his family.

The Medical Department is not willing to be accorded a second place in comparison with any other arm of the service. It points with pride to its roll of honor during the last war, to the 38 of its members who were killed in action or died of wounds, to the 12 who were killed by accident in the performance of duty, to the 4 who died as prisoners of war, to the 73 who were wounded in battle, and to the 271 who died from the diseases and exposure of camp life (an array of figures greater than can be presented by any other staff corps), as a proof that they were always to be found where duty called."

It is believed that at least as high a grade of intellect, as finished an education, as eminent scientific attainments are required to make an accomplished medical officer as obtains in any other branch of the service. In the case of the vast majority of other staff officers, this education is obtained at the expense of the government, which not only furnishes the future Adjutant-General or Engineer with all the advantages which a scientific school can afford, but actually pays him a handsome salary for four years before he is sufficiently accomplished to render any service therefor. Not so the medical officer. He must obtain his education at his own expense, usually spending years in college, in medical schools, and hospitals, before he can hope to be sufficiently qualified to pass the ordeal of an examining board.

The rank of medical officers should, therefore, be no less than that of the other staff corps; they require an equal education and equal abilities, they perform equally arduous duty, they sustain as great responsibilities, they are exposed to like dangers, and in the distribution of the rewards of the military career, they should be entitled to an equal share. That they are not at present on this footing of equality has been clearly shown in the foregoing comparisons.

A further injustice has been done, nct only to the Medical Staff, but to the profession at large, by the legislation which at present forbids any promotions or new appointments in the Medical Department. To the former, because, from the rapid depletion which ordinary casualties make in its ranks, it throws incre: sed labor upon those who remain, and necessitates the employment of physicians under contract, who, having but a temporary tenure of office, cannot be expected to be actuated by that single regard for the service, which is a *sine qua non* to the faithful performance of duty. To the profession at large, for it prevents many who are desircus of entering the military service from doing so, and thus perhaps compels them to abandon a design to the accomplishment of which their education may have been especially directed."

The letter sets forth the fact that there were sixty-one vacancies in the staff, fifty-three of them being in the grade of Assistant Surgeon. Unless some encouragement in the shape of a hope of promotion is held out to medical men few will be induced to enter the service, and the consequence will be that an increased amount of labor will be thrown upon those already in the service. The medical staff of the U. S. Army is second to none in the world in its devotion to duty and the advancement of medical science. It contains men in its ranks who would adorn any position in the profession, and the medical profession of the United States look with pride upon the contributions to scientific and practical medicine and surgery which have emenated from this source. In their devotion to duty they have sacrificed the comforts of home, health and even life itself, and their brethren in the profession demand that a suitable recognition be made of their services. This recognition can in a measure be made in the manner asked, namely an increased rank in the service, so that the medical officer may have something to look forward to after years of arduous service. This increase of rank has already been granted to the medical staff of the Navy, and no good reason exists why it should not be extended to that of the army.

We hope that Congress will take immediate action in the case.

The following is a copy of the proposed law:

### A BILL

*To Increase the Efficiency of the Medical Department of the Army.*

SECTION I—Be it enacted by the Senate and House of Representatives of the United States of America, in Congress assembled, That so much of the sixth section of the act entitled, "An Act making appropriations for the support of the Army and for other purposes," approved March 3, 1869, as forbids promotions and appointments in the medical department of the army be, and the same is hereby, repealed.

SEC. II.—And be it further enacted, That the organization of the medical department shall be as authorized in the seventeenth section of the act entitled, "An act to increase and fix the Military Peace Establishment of the United States, approved July 29, 1866. *Provided*, that from and after the passage of this Act, the Chief Medical Purveyor and four Assistant Medical Purveyors, now authorized by law, shall have the rank, pay, and legal allowances of Colonels; and *Provided, further*, that surgeons who have served thirty years and upward, from the date of their original entry into service, shall have the rank, pay, and legal allowances of Colonels, and surgeons who have served less than thirty and more than twenty years, from the date of their original entry into service, shall have the rank, pay, and legal allowance of lieutenant colonels, and all other surgeons shall have the rank, pay, and legal allowances of Majors, as now authorized by law, and the rank, pay, and legal allowances of assistant surgeons shall be as now authorized by law. *And provided further*, that the foregoing provisions of this Act shall apply to all officers who may hereafter be promoted in the medical department.

SEC. III.—And be it further enacted, That nothing in this act shall be construed to permit the appointment or promotion of any person in the medical department until he shall have passed the examination now required by law.

SEC. IV.—And be it further enacted, That all laws and parts of laws inconsistent with the provisions of this Act be, and the same are hereby, repealed.

